# Research cardiac magnetic resonance imaging in end stage renal disease - incidence, significance and implications of unexpected incidental findings

**DOI:** 10.1007/s00330-016-4288-4

**Published:** 2016-04-07

**Authors:** Elaine Rutherford, Jonathan R. Weir-McCall, Rajan K. Patel, J. Graeme Houston, Giles Roditi, Allan D. Struthers, Alan G. Jardine, Patrick B. Mark

**Affiliations:** 1 Division of Cardiovascular and Diabetes Medicine, Ninewells Hospital, Dundee, DD1 9SY UK; 2 Institute of Cardiovascular and Medical Sciences, British Heart Foundation Building, Glasgow Clinical Research Centre, Glasgow, G12 8TA UK; 3 Department of Radiology, Glasgow Royal Infirmary, NHS Greater Glasgow and Clyde, 84 Castle Street, Glasgow, G4 0ET UK

**Keywords:** Chronic kidney disease, Clinical trials, MRI, Incidental findings, CMR

## Abstract

**Objectives:**

Left ventricular mass (LVM) at cardiac magnetic resonance imaging (CMR) is a frequent end point in clinical trials in nephrology. Trial participants with end stage renal disease (ESRD) may have a greater frequency of incidental findings (IF). We retrospectively investigated prevalence of IF in previous research CMR and reviewed their subsequent impact on participants.

**Methods:**

Between 2002 and 2006, 161 ESRD patients underwent CMR in a transplant assessment study. Images were used to assess LV mass and function. In the current study a radiologist reviewed the scans for IF. Review of patient records determined the subsequent clinical significance of IF.

**Results:**

There were 150 IF in 95 study participants. Eighty-four (56 %) were new diagnoses. One hundred and two were non-cardiac. Fifteen were suspicious of malignancy. There was a clinically significant IF for 14.9 % of the participants. In six cases earlier identification of an IF may have improved quality of life or survival.

**Conclusions:**

Without radiology support clinically important IF may be missed on CMR. Patients undergoing CMR in trials should be counselled about the frequency and implications of IF. Patients with ESRD have a higher prevalence of IF than reported in other populations. Nephrology studies require mechanisms for radiologist reporting and strategies for dealing with IF.

***Key Points*:**

*• Incidental findings on research cardiac magnetic resonance imaging can have significant consequences.*

*• We considered incidental findings in historical renal cardiac resonance imaging clinical trials.*

*• Incidental findings are common and important in the chronic kidney disease population.*

*• Without radiology support, clinically significant incidental findings may be missed on imaging.*

*• Study protocols, approvals and consent processes should take account of possible findings.*

## Introduction

Reduction in cardiac morbidity and mortality is of great importance in nephrology. Within the chronic kidney disease (CKD) population, increased left ventricular mass (LVM) is well established as a surrogate marker of cardiovascular risk [1]; therefore, reduction in LVM assessed by cardiac magnetic resonance imaging (CMR) is a frequent primary end point in nephrology clinical trials [2–9].

Patients consenting to participate in clinical trials undergo CMR that would not otherwise be performed. Standard CMR imaging techniques commonly capture windows out with the area of the heart including the abdomen and thorax. Clearly incidental findings (IF) on images obtained for research may have unexpected clinical consequences. The prevalence of unexpected findings in clinically indicated CMR has been well documented [10–13]. While the reported incidence of incidental non-cardiac findings in non-renal populations varies from around 7-27 %, the rate of clinically significant IF is much lower than this at around 1-7 % [10–13]. The rate of IF in CMR performed for research purposes is less well established [14]. However, findings in research CMR are important and current radiology guidelines reflect this [15].

Trial participants with CKD may be at increased risk of having clinically significant IF. In those with end stage renal disease (ESRD) the risk may be even greater as these individuals are likely to have a larger burden of ill health. This potential increased risk of IF could have ethical, clinical and financial implications for future CMR nephrology studies and their participants. The prevalence of IF in CMR in patients with ESRD requiring dialysis is unknown. To investigate this, we retrospectively determined the prevalence of IF in previous research CMR and reviewed its potential subsequent impact on patient care.

## Subjects and methods

### Participants

Between 2002 and 2006, 161 patients underwent CMR as part of a prospective single centre research study considering cardiac risk and transplantation assessment under the care of the Renal Transplant Unit at the Western Infirmary Glasgow [8, 9, 16, 17]. All participants had CKD stage 5 (estimated glomerular filtration rate < 15/min) and were receiving peritoneal or haemodialysis, or were within 6 months of requiring it (pre-dialysis). Participants had no contraindications to CMR but were otherwise an unselected group. The study was approved by the local ethics committee. Prior to imaging, all participants provided written informed consent.

### CMR acquisition and analysis

CMR imaging was performed using a 1.5-Tesla magnetic resonance imaging scanner (Sonata, Siemens Erlangen, Germany). In the original study, images were used by our research group solely to assess cardiac parameters, in particular LVM and cardiac function. For those on haemodialysis, the scans were consistently performed 24 hours after the end of the last dialysis session. Following acquisition of localizer images using a gradient echo sequence (FLASH: TR = 3.25 ms; TE = 1.6 ms; FA = 25^0^; pixel area = 1.4 mm by 1.4 mm; slice thickness = 8 mm; FOV = 360 mm) a steady-state free precession (TrueFISP) sequence was used to acquire cine images in three long axis planes (vertical long axis, horizontal long axis, left ventricular outflow tract) followed by short axis sequences from the atrioventricular ring to the apex. Cine sequences were obtained with the following acquisition parameters: TR = 3.14 ms; TE = 1.6 ms; FA = 60^0^; pixel area = 2.2 mm by 1.3 mm; slice thickness = 8 mm; FOV = 340 mm. Evidence of prior infarcts was sought on short axis late gadolinium enhancement sequences. All these studies were performed prior to any link between the use of gadolinium contrast agents with nephrogenic systemic fibrosis (NSF) was established in CKD patients [18, 19]. Late gadolinium enhancement imaging was acquired using a breath-hold segmented turbo fast low angle shot (FLASH) inversion-recovery sequence with the following acquisition parameters: TR = 11.6 ms; TE = 4.3 ms; FA = 20^0^; pixel area = 2.2 mm by 1.3 mm; slice thickness = 8 mm; FOV = 340 mm. From March 2004, in order to assess aortic indices, a transverse HASTE stack of the thorax was adopted into our imaging protocol (TR = 600 ms; TE = 24 ms; FA = 160^0^; pixel area = 1.4 mm by 1.4 mm; slice thickness = 8 mm; FOV = 360 mm). From this a ‘candy cane’ TrueFISP sequence of the thoracic aorta was obtained. An axial oblique through plane phase contrast sequence was also obtained through the thoracic aorta at the level of the right pulmonary artery. This was performed in 113 patients. In the current study, an experienced radiologist from another centre reviewed all images for IF. The reviewing radiologist was blinded to patient identity, outcomes, original scans and subsequent imaging.

### IF classification

Findings were categorized as cardiac and non-cardiac IF. Cardiac findings were sub-classified as valve, pericardial or findings suggestive of cardiomyopathy. Multiple valve abnormalities on a scan were considered a single finding. Abnormalities in ventricular mass, size, perfusion or function were not considered IF unless consistent with a previously undiagnosed non-uraemic cardiomyopathy.

Non-cardiac findings were subcategorized as lung/mediastinal, pleural effusions, gastrointestinal, hepatic/renal cysts and ‘other’ findings. Renal atrophy was not considered an IF. Hepatic and renal cysts were not considered IF, if there was a history of polycystic kidney disease.

### Establishing clinical significance

**Fig. 1:**
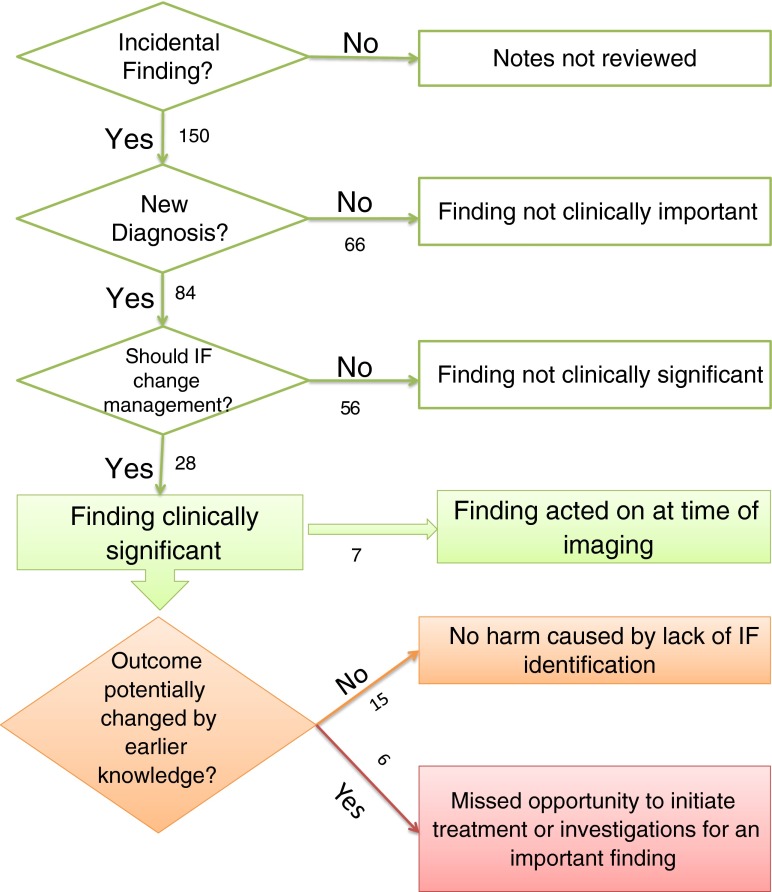
Flow diagram showing the number and significance of findings at each level of importance. The numbers represent the number of findings at each point in the process

Current and historical electronic clinical and radiology records for all participants with one or more IF were reviewed by a clinician who was not involved in subsequent patient care. The end of the follow-up period for establishing IF significance was considered as the date of death or the date clinical records were reviewed (if later). Whether a finding raised the possibility of malignancy was noted. Figure 1 details the process followed for determining the significance of IF. The clinical significance of IF was determined by answering three questions:

- *Did the finding represent a new diagnosis?*
  Cases where it was not possible to determine if the finding represented a new diagnosis or not were not considered new diagnoses. Pleural effusions were only considered to be a new diagnosis if they were unilateral and at least moderately sized.
- *Did, or would, the finding have altered clinical management at the time of imaging (e.g., further investigation or referral)?*
  IF were considered significant if they altered, or would have altered patient management when recognised.
- *Would earlier knowledge of the finding have feasibly altered the patient’s clinical course and potentially improved quality of life or survival?*
  This question was only asked of significant IF.

### Statistics

Statistical analysis was performed using SPSSv21 (IBM, Armonk, NY, USA). For normally distributed data, a t-test was used to compare means across groups. For non-parametric data, a Mann-Whitney test was used. Regression analysis determined whether the presence of an IF was predictive of poorer survival.

## Results

### Participant characteristics

Mean age at time of imaging was 52.3 years (range 25-77 years). A full breakdown of baseline patient demographics and available clinical data is available in Table 1. As in our original research, there was a high prevalence of traditional cardiovascular risk factors [9]. At the end of follow-up, 50.3 % of participants were alive. Median follow-up duration was 10.0 years (range 1 day-12.4 years).

**Table 1:** Baseline demographic and clinical data for participants

Variable	Total (161 participants^a^)
Age at CMR (years)	52.3
Male	104 (64.6 %)
Body surface area (m^2^)	1.84 (±0.23)
BMI (kg/m^2^)	26.02 (±5.07)
Systolic blood pressure (mmHg)	138 (±25)
Diastolic blood pressure (mmHg)	82(±13)
Renal replacement therapy	
Haemodialysis (%)	44.7
Peritoneal dialysis (%)	35.4
Pre-dialysis (%)	19.9
Diabetes mellitus (%)	31
Hypertension (%)	50.6
Previous myocardial infarction (%)	5.7
Ischaemic heart disease (%)	8.2
Cerebrovascular disease (%)	4.4
Peripheral vascular disease (%)	2.5
Dyslipidaemia (%)	24.7
Never Smoker (%)	53.2
Current/ Ex smoker (%)	46.8
Haemoglobin (g/dL)	11.45 (± 1.97)
Corrected calcium (mmol/l)	2.29 (±0.31)
Albumin (g/dl)	39.2 (±4.8)
Phosphate (mmol/l)	1.68 (±0.5)
CMR ejection fraction (%)	67.5 (±11.2)
CMR left ventricular mass index (g/ m^2^)	91.4 (±27.6)

^a^Full clinical demographic data was not available for 5 participants

Participants with an IF tended to be older than those without, although this was not statistically significant (mean 53.6 years vs 50.4 years, respectively, *P* = 0.08). There was no difference in prevalence of IF across modality of renal replacement therapy or any other baseline characteristic.

### Number and significance of findings

Review of the 161 CMR scans revealed 150 IF in 95 patients (59 % of patients). Eighty-four (56 %) of the IF in 66 patients would have resulted in a new diagnosis.

There were 28 significant IF that warranted further investigation or a change in patient management. Twenty-four participants had one or more significant findings (14.9 % participants). Seven significant IF were identified and acted on at the time of imaging. There were 21 cases of potentially significant IF where the finding was not identified at the time of imaging. In 15 of these cases, record review confirmed that lack of intervention ultimately did not harm patient outcome. In six cases, affecting four (2.5 %) participants, lack of recognition of the finding was a missed opportunity to initiate investigations or treatment for a finding that later became clinically significant. Earlier identification of these findings may have improved the patients’ quality of life or survival (Table 2). Figure 1 illustrates the process of determining the significance of the IF and the number of findings at each level of significance.

**Table 2:** Summary of incidental findings where earlier detection may have improved quality of life or survival

Finding on CMR	CMR year	Comment
Hypertrophic obstructive cardiomyopathy	2004	Eventually diagnosed 2011 following progression of symptoms
Lung lesion highly suspicious of malignancy^a^ (Fig. 5)	2004	Died of possible malignancy 2008
Oesophageal lesion highly suspicious of malignancy^a^(Fig. 5)	2004	Died of possible malignancy 2008
Large unilateral pleural effusion	2004	Lower lobe lobectomy for presumed adenocarcinoma 2007
Multiple suspicious splenic lesions^b^ (Fig. 4)	2004	Picked up incidentally on abdominal CT 2013 – resulted in 6 month unnecessary suspension from transplant list
Multiple suspicious liver lesions^b^	2004	Picked up incidentally on abdominal CT 2013 – resulted in 6 month unnecessary suspension from transplant list

^a^lesions were in same participant. Cause of death on death certificate was ‘cardiac arrest’ but participant’s clinical team felt malignancy may have been the actual cause of death – post mortem was not pursued

^b^lesions were in same participant

### Influence of imaging protocol on IF rate

There was no difference in the rate of IF in those who had a HASTE stack performed as part of their imaging sequence and those who did not. Contrast administration was important for the late gadolinium enhancement IF, where it contributed to the diagnosis of Hypertrophic Obstructive Cardiomyopathy (HOCM). In an additional case, perfusion sequences were useful to characterize a hepatic lesion, which was indeterminate on other sequences, but showed classical enhancement of a haemangioma. Other than these scenarios, contrast added little to the diagnostic yield.

### Cardiac findings

There were 48 cardiac IF, 34 (70.8 %) were new diagnoses. Of these, nine (26.5 %) did, or would have warranted a change in patient management when recognized. On one occasion earlier identification of a finding of HOCM may have led to an earlier change in the patient’s treatment and potentially improved quality of life.

### Valve findings

Valve disease was the most common cardiac IF (34 cases, 70.8 % of cardiac findings). Table 3 gives a breakdown of which valves were affected. Eleven findings (32.4 %) of valve disease were known about prior to CMR imaging. Valve disease was a new diagnosis in 23 cases, in 15 (65.2 %) of these cases, there was clear documentation that the abnormality was identified at the time of scanning. In nine of these 15 cases, no changes to subsequent patient management were necessary. In the remaining six cases, the diagnosis had a direct impact on patient care (e.g., referral for valve replacement).

**Table 3:** Breakdown of type of incidental valve lesions

Valve lesion	Number of participants with findings^a^
Aortic regurgitation	9
Aortic stenosis	22
Mitral regurgitation	10
Tricuspid regurgitation	5

^a^12 participants had more than one valve lesion

There were eight cases where there was no specific mention of valve disease on available documentation. In seven of these cases, it was clear from note review that these lesions would not have significantly altered subsequent patient management. In a single case, knowledge of the valve disease – severe aortic stenosis – would have changed management, but there was in practice insufficient time between scanning and the patient’s death (less than 48 hours) for any changes to be effected.

### Cardiomyopathy

The presence and characteristic findings of uraemic cardiomyopathy in this population group have been well described with Left Ventricular Hypertrophy (LVH) and dilatation being prominent findings [9, 20]. The reviewing radiologist considered that ten participants had changes that could be broadly consistent with a cardiomyopathy. Changes consistent with cardiomyopathy accounted for 6.7 % of all IF and affected 6.2 % of the study population. In four cases this was already known about prior to CMR. In six cases this represented a new diagnosis, in two of these new cases, potential cardiomyopathy identification changed immediate patient management. In one case, the findings were noted but no changes to patient management were appropriate. In three cases, no comment was found in historical records with regard to cardiomyopathy. Patient outcome could conceivably have been influenced in only one of these cases. In this case, the radiologist considered a 2004 scan to be consistent with HOCM – our research group interpreted these changes as showing evidence of prior ischaemia and LVH. In 2011 this patient was diagnosed with HOCM on repeat CMR following progressive symptoms consistent with the diagnosis. Although HOCM is a progressive condition, better symptom control could arguably have been achieved through earlier recognition of this IF. However these types of finding are clearly subject to interpretation in the context of uraemic cardiomyopathy, which has many features that may overlap with ‘early’ HOCM.

### Non-cardiac findings

There were 102 non-cardiac IF affecting 43.5 % of participants. Fifty non-cardiac findings (49.0 %) were classed as new diagnoses. Of these 50, 19 (38.0 %) warranted further investigation or a change in patient management when identified. On five occasions, lack of recognition of such a finding was a missed opportunity to initiate further investigations or treatment for a finding that later became clinically significant (Table 2). Earlier identification of these findings may have led to a change in the patient’s clinical course and potentially improved quality of life or survival. Table 4 gives a breakdown of the different categories of non-cardiac findings and their ultimate impact on patient care.

**Table 4:** Breakdown of the number of different types of non-cardiac findings and their impact on patient care

Finding classification	Total number of findings	New diagnosis	Would have changed management if identified but did not alter clinical course	Identification could feasibly have altered clinical course
Lung or mediastinal finding	11	9	6	1
Pleural effusion	15	4	3	1
GI tract	19	12	1	3
Hepatic/Renal cyst (Not known PKD)	50	18	1	0
Other significant finding	7	7	3	0
Total	102	50	14	5

### Findings suggestive of malignancy

All of the 14 non-cardiac IF that would have triggered further investigation, but ultimately did not alter the participant’s clinical course, were suspicious of malignancy. In total there were 15 IF that were suspicious of malignancy. These IF and patient outcomes are detailed in Table 5.

**Table 5:**
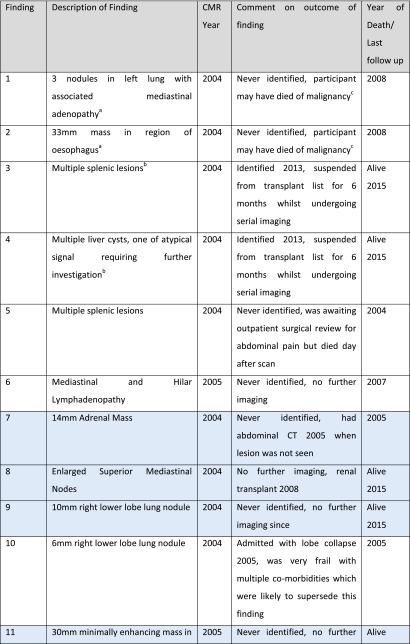

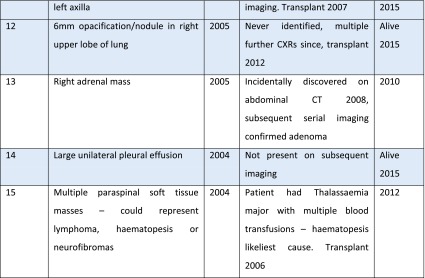
Summary of incidental findings potentially suspicious of malignancy

### Influence of IF on length of survival

**Fig. 2:**
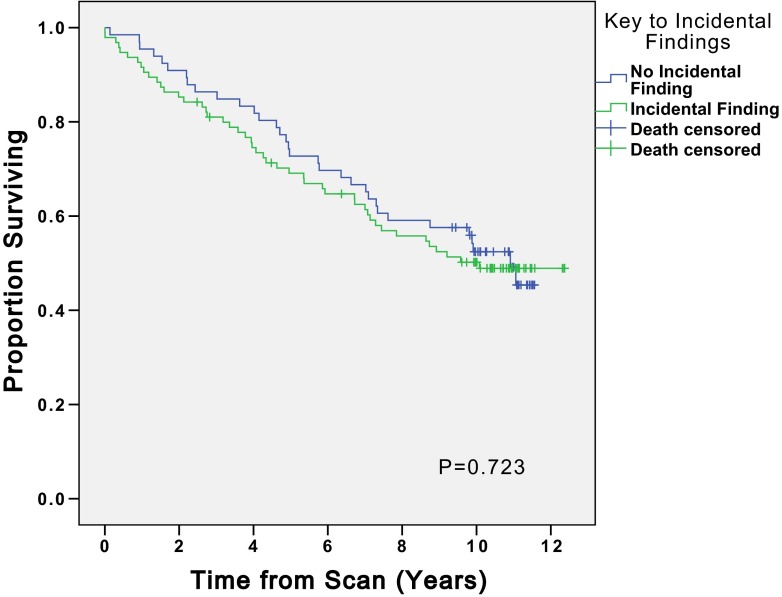
Kaplan Meier plot showing comparison of survival in those with and without any incidental findings

The presence or absence of an IF on imaging did not have any statistically significant impact on length of survival. See Fig. 2 – Kaplan Meier Death-censored survival. Estimated mean survival: No IF 8.4 (95 % CI 7.5-9.3) years vs with an IF 8.3 (95 % CI 7.4-9.2) years.

## Discussion

**Fig. 3:**
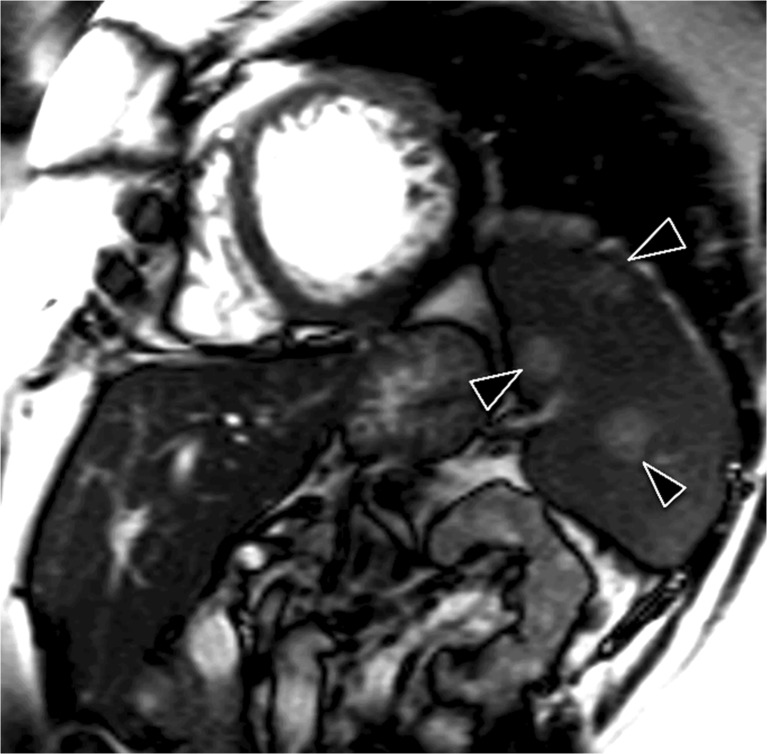
Multiple high signal splenic lesions (arrow heads) are visible on this short axis cine. No prior history of malignancy was present

This is the first study looking at the rate of IF on CMR performed for research purposes in the ESRD population. Our study highlights that IF in research CMR in this population are both common and have potentially serious clinical implications (Fig. 3). The prevalence of IF in our study population is greater than that in several studies looking at the rate in clinically indicated CMR [10, 11, 13]. In fact, it is more than seven times greater than the rate reported by Chan in a clinically robust study of 1534 consecutive clinically indicated CMR studies (59.0 % vs 7.6 %) [10]. This is striking as Chan’s group was examining clinically indicated CMR images and one might expect the prevalence of IF in clinically indicated scans to be considerably higher than that of non-clinically indicated research scans. As our scans were undertaken purely for research purposes, we also included a limited number of cardiac IF in our initial analysis. However, even if cardiac IF are excluded, the prevalence of non-cardiac IF in our study population (43.5 %) is higher than a number of previously reported rates (range 7.6 %-27 %) [10–13, 21].

Only one major study has a rate of IF significantly higher than ours. This study was performed specifically to determine the rate of IF on CMR in 132 volunteers revealed IF in 81 % of participants [14]. However, this remarkably high prevalence rate is not truly comparable to ours as this study classed a much broader range of findings as IF (for example sternotomy wires). Their participants were also considerably older (mean age 74.2). Furthermore their study did not consider the subsequent real life clinical significance of their findings.

**Fig. 4:**
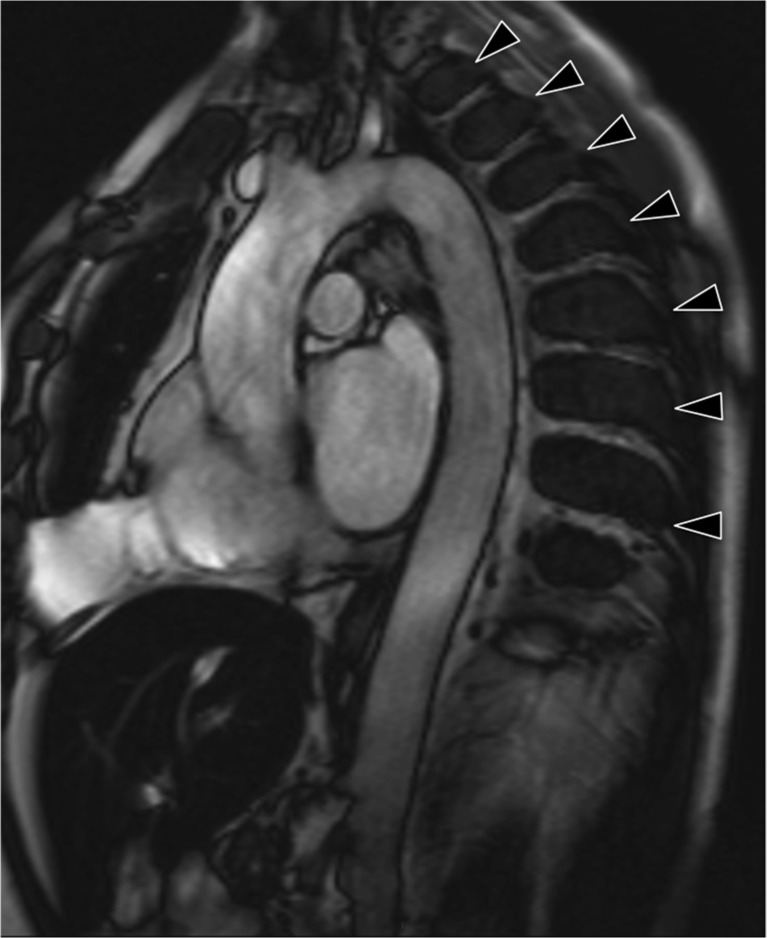
Multiple regularly spaced low signal paraspinal masses (arrow heads) are evident on this candy cane view of the aorta. Note also the low signal within the liver, the combination of which is consistent with extramedullary haematopoesis in a patient with thalassaemia

Not only was our rate of finding higher than in comparable studies, our study revealed a large number of clinically significant findings. Review of our images resulted in 84 new diagnoses in 161 studies. However, we have not considered all new diagnoses to be clinically significant. Whilst arguably useful to know about, asymptomatic diverticulosis or gallstones is unlikely to have any significant impact on any individual (Fig. 4).

Of much more importance to trial participants is if any IF would lead to further investigation, or to a change in their management. Like Sohns [13], we chose to classify findings as significant or not on this basis. Using this more stringent definition of a significant finding, 14.9 % of our participants had a significant new IF. This is higher than in some other clinical studies where comparable definitions have been used (range 0.4-7 %) [10–13]. Given such a high prevalence rate, it is our recommendation that all potential participants in renal studies involving CMR are counselled about the possibility of significant findings and the impact that they could have on their care prior to obtaining consent.

Uniquely in this study, follow-up of all patients with IF through review of electronic records allowed us to determine the subsequent true impact of these significant findings on patient care. In seven cases identification of a major finding at the time of imaging directly led to a significant change in patient management (for example, aortic valve replacement). Such cases may partially explain why there was no difference in survival of those with and without findings – the actions taken as a result of a major finding at the time of imaging may have improved subsequent participant survival (Fig. 2). However, the main reason why we saw no difference in survival of the two groups is likely to be due to the confounding multiple co-morbidities of the ESRD population.

**Fig. 5:**
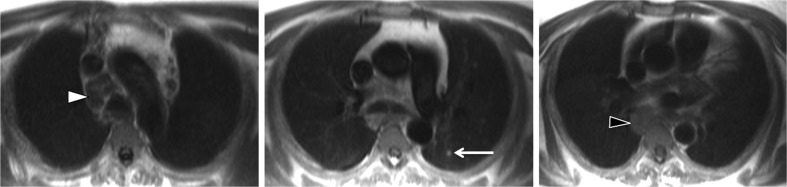
Oesophageal mass/para-oesophageal adenopathy (black arrow head), upper mediastinal nodes (white arrow head) and lung nodules (arrow)

At an individual level there were cases where survival may have been influenced had the images been reported at the time of imaging. For four participants (six significant findings) the abnormality was not identified at the time of imaging and an opportunity to influence patient care at an earlier stage was missed (Fig. 5). Those six findings are detailed in Table 1. In order to ensure such opportunities are not missed in future renal studies, we recommend that all research images obtained should be reported by a suitably trained radiologist within clinically acceptable timeframes. This is in keeping with current radiology research guidelines [15].

We also wish to point out that early knowledge of IF is not always an advantage. Table 5 highlights findings that could be interpreted as being suggestive of malignancy, the findings highlighted in blue were not identified at the time of imaging. Follow up of these participants revealed that the potentially suspicious finding did not impact on their clinical course. Such findings are incidentalomas and their discovery at the time of imaging may have caused considerable stress and subjected participants to unnecessary further investigations. Subjects could even have been harmed by IF identification, as a number of the findings would have resulted in arguably unnecessary transplant listing suspension until investigations were completed. This potential risk of harm should also be detailed to potential study participants prior to obtaining their consent. However, the authors of this paper believe the potential benefits of early identification of potentially significant IF far outweigh the potential risks.

Given the importance of IF within this research population, each study should have defined protocols for how IF will be managed. Ethical committees considering prospective approval of renal research studies should consider the implications of IF and ensure that there is a robust protocol for their management prior to granting study approval.

It is worth highlighting that the burden of IF in research CMR in this population is also likely to be replicated in CMR performed for clinical reasons. Current guidelines recommend that all individuals, regardless of presence or absence of symptoms, should have an echocardiogram shortly after commencing renal replacement therapy [22]. We know that increased LVM is strongly predictive of outcome in all cases, including those with ESRD [1, 23]. A number of studies have shown that LVM measurement by echocardiography in this population is less robust than by CMR [24, 25]. As the cost of CMR falls and it is more widely available, its role in the clinical assessment of ESRD patients is likely to expand. Future guidelines may include assessment of LV indices with CMR. This means the frequency of incidental findings in this population will become more relevant as CMR is increasingly used for baseline clinical cardiac investigations.

Finally, the link between gadolinium based contrast used for MRI imaging and development of NSF in patients with advanced CKD, means that this study cannot be reproduced [19, 26]. Whilst we did not find gadolinium contrast necessary in all cases to delineate the nature of an IF, it is likely that further studies in the ESRD population may require follow-up imaging with computed tomography with iodinated contrast to follow up on indeterminate findings of non-contrast CMR.

### Limitations

Our study is a retrospective study using historical images. Given the number and significance of findings discovered, a prospective study would be ethically entirely inappropriate.

Undoubtedly methods of determining what constitutes an IF will vary slightly between studies and reporters, and it has not been possible to adjust for this.

In studies such as ours, where a further focused reading of the CMR images has taken place, it might be expected that the rate of findings would be slightly greater than in other studies. For example, Wyttenbach [27] found the rate of significant IF identified increased from 11.7 % to 20.5 % when the same clinician re-interpreted historical clinical scans specifically looking for extra-cardiac IF. However, this does not detract from the overall value of this study, which highlights that IF in research CMR in the ESRD population are both common and important.

## Conclusions

This study of 161 CMR images performed for research purposes in the CKD5 population revealed 150 IF. 14.9 % of the participants had a new significant IF that warranted further investigation or a change in their management.

The prevalence of IF in the ESRD population is such that all investigators planning studies undertaking CMR must take proper account of it. All investigators should consult with experienced radiologists early in the trial planning phase to ensure an appropriate mechanism for reporting is in place. Funding for radiologist reporting should be factored into grant applications. All images obtained should be reported by a qualified radiologist within a clinically acceptable timeframe.

All potential participants in renal CMR research studies should be counselled about the frequency and implications of possible IF and protocols for managing IF should be part of good clinical research practice.

## Abbreviations

CKD: Chronic kidney disease
CMR: Cardiac magnetic resonance imaging
ESRD: End stage renal disease
HOCM: Hypertrophic obstructive cardiomyopathy
IF: Incidental findings
LVH: Left ventricular hypertrophy
LVM: Left ventricular mass
